# Application of Amplicon Metagenomics to Identify Fungal Pathogens in Formalin-Fixed Paraffin-Embedded Samples: Proof of Concept in Animals with Fungal Pathologies

**DOI:** 10.3390/microorganisms13030533

**Published:** 2025-02-27

**Authors:** David B. Needle, Guillaume Reboul, Patrick K. Mitchell, Derek Rothenheber, Nicholas J. Marra, Brittany D. Cronk, Neeti G. Patel, Laura B. Goodman

**Affiliations:** 1 New Hampshire Veterinary Diagnostic Laboratory, College of Life Sciences and Agriculture, University of New Hampshire, Durham, NH 03824, USA; 2 Department of Public and Ecosystem Health, College of Veterinary Medicine, Cornell University, Ithaca, NY 14853, USA; gcr58@cornell.edu (G.R.); ngp39@cornell.edu (N.G.P.); 3 Animal Health Diagnostic Center, College of Veterinary Medicine, Cornell University, Ithaca, NY 14853, USA; mitchell.patrick.k@gmail.com (P.K.M.); derek.rothenheber@gmail.com (D.R.); bdc72@cornell.edu (B.D.C.); 4 Division of Science, Mathematics and Technology, Governors State University, University Park, IL 60484, USA; nmarra@govst.edu

**Keywords:** fungal diagnosis, molecular diagnostics, metabarcoding, FFPE sequencing, bioinformatics

## Abstract

The identification of fungal pathogens in formalin-fixed paraffin-embedded (FFPE) tissues is an unmet need in human and animal medicine, and sequence-agnostic approaches are needed to identify emerging pathogens. Eleven FFPE biopsy specimens with etiologic diagnoses of fungal disease based on standard testing of paired fresh tissue samples were utilized here to evaluate metabarcoding approaches. The cases included tissues from three dogs, three cats, one box turtle, one goat, one common loon, and one gray tree frog. The diagnoses from the fresh tissues in these cases were *Microsporum canis*, *Penicillium* sp., *Exophiala* sp. (likely *E. jeanselmei*), *Verticillium* sp., *Rhizopus* sp., atypical *Cryptococcus neoformans*, *Conidiobolus* spp., *Aspergillus fumigatus*, *Cryptococcus neoformans var grubii*, *Batrachochytrium dendrobatidis*, *Fusarium solani*, *Blastomyces dermatitidis*, *Coccidiodes immitis*, and *Histoplasma capsulatum*. We compared the ITS1 and 28S D1 rRNA gene genetic markers in combination with several bioinformatic strategies to identify fungal pathogens in the FFPE tissue samples, with a success rate of 9/11. These methods could allow diagnosticians who receive only FFPE tissues and see fungal pathogens to speciate the pathogens and could be of value in retrospective studies wherein FFPE tissue is the only archived tissue. Furthermore, these techniques could be of use to researchers investigating polymicrobial communities where DNA preservation is suboptimal.

## 1. Introduction

### Importance

Skin biopsies and other tissues collected for microscopic observation are typically put into formalin to preserve the relevant structures for the pathologist. When evidence of a fungal pathology is seen, it is then very difficult to determine the fungal species if a fresh sample was not also preserved. Formalin fixation not only prevents culture but also makes direct molecular detection more challenging due to DNA damage. Here, we provide proof of concept of a new strategy to combine the amplification of two genetic markers for fungal typing with benchtop sequencing using equipment becoming increasingly available in clinical microbiology laboratories.

Over the past several decades, fungal infections have risen to threaten population health across large regions of multiple species, including amphibians, reptiles, mammals, and humans. Fungal infections have been tied to altered climates and globalization, with emergence and disease associated with increased ambient temperatures and humidity [1,2]. Of note are *Cryptococcus neoformans* infections associated with HIV; *Batrachochytrium dendrobatidis*, decimating frog populations worldwide since 1998; snake fungal disease caused by *Ophidiomyces ophiodiicola* in snakes in North America and Europe; *Pseudogymnoascus destructans*, inducing white-nose syndrome in North American bats; and dermatophytosis in humans due to *Trichophyton mentagropyhtes* across the Indian subcontinent [3,4,5,6,7]. Each of these fungal diseases has had large-scale, population-wide effects. In humans, the independent emergence of multidrug-resistant and environmentally stable *Candida auris* in three different continents has raised alarm as being the first fungal species with pandemic potential [8]. The initial challenges in the identification of this species in clinical samples highlighted the need for sequence-based approaches that can be implemented rapidly in a species- and sample-independent manner [9].

According to the Fungal Diagnostics Laboratories Consortium, sequencing for the direct identification of fungi in formalin-fixed paraffin-embedded (FFPE) tissue samples is a major diagnostic capability gap that should be prioritized for optimization [10]. In both human and animal medicine, fresh biopsy samples are often not saved for potential culture, as fungal pathogens may not be suspected until the biopsies are read. Although FFPE tissues are an ideal archival sample for histopathology and immunohistochemistry, formalin fixation cross-links DNA molecules, which reduces the length of fragments available for amplification and sequencing. There is no universal marker for fungi of equivalent size and utility to the bacterial 16S, which allows a good resolution with shorter (<600 bp) segments with Illumina chemistry. FFPE samples are acceptable for PCR with such relatively short target sequences but are, generally, not ideal samples for fungal metagenomics using single-marker barcoding and long-read (Sanger or Oxford Nanopore) chemistry. Sequencing multiple short amplicons for metagenomics with Illumina sequencing offers the prospect of obtaining and identifying sequences from a relatively low number of fragmented initial targets, as would be expected in FFPE samples.

In collaboration with other investigators, we recently characterized the infection of twelve free-ranging, wild porcupines with *Trichophyton benhamiae*, resulting in severely debilitating and fatal dermatophytosis for those untreated [11]. As part of that work, we applied next-generation sequencing to archived formalin-fixed tissue samples, a novel metagenomic approach that identified commensal fungi as well as *Trichophyton* spp. This result spurred us to hypothesize that the application of similar and further developed techniques could be effective in identifying different fungal pathogens in FFPE tissues. Here, we present the application of next-generation sequencing to perform amplicon metagenomics on archived FFPE tissues from clinical cases in eleven animals containing fungal pathogens that were identified previously via histology, PCR, and sequencing in fresh tissue or culture samples.

## 2. Materials and Methods

### 2.1. Cases with Fungal Pathologies

No research on animals was performed. Eleven fungal cases were included in this study. To produce a series of cases with varied fungal pathogens, a retrospective analysis of biopsy and necropsy cases in the archives of the New Hampshire Veterinary Diagnostic Laboratory was performed to identify cases with diagnosed fungal disease as part of routine clinical care (see Table 1 and Table 2 for the specific diagnoses and agents). A further inclusion criterion was confirmation of the infectious fungal disease by an alternate method. This included molecular testing in fresh tissue (case 10), histopathology (cases 5 and 11), or purified cultured fungi identified by MALDI-TOF (all other cases). The confirmatory PCR and sequencing in case 10 were performed as a referral test at the Wisconsin Animal Disease Diagnostic Laboratory. The case information, including initial routine diagnostic results, is summarized in Table 1. The host species included three dogs (*Canis familiaris:* cases 1, 9, and 10), two cats (*Felis catus*: cases 3 and 6 (same cat), and 11), one common box turtle (*Terrapene carolina*: case 2), one goat (*Capra aegagrus hircus*: case 4), one common loon (*Gavia immer*: case 5), one gray tree frog (*Hyla versicolor*: case 7), and one red-footed tortoise (*Chelonoidis carbonaria*: case 8). The lesions affected cutaneous tissues (including the shell carapace and plastron in one tortoise) in seven animals, the respiratory tract in the goat and the loon, a peripheral lymph node in one dog, and the soft tissue of the body wall in the red-footed tortoise.

### 2.2. Testing of Fresh Tissues

The confirmed organisms in order of case number were (1) *Microsporum canis*; (2) *Penicillium* sp., *Exophiala* sp. (likely *E. jeanselmei*), *Verticillium* sp., and *Rhizopus* sp.; (3) *Cryptococcus neoformans*; (4) *Conidiobolus* sp.; (5) *Aspergillus fumigatus*; (6) *Cryptococcus neoformans var grubii*; (7) *Batrachochytrium dendrobatidis*; (8) *Fusarium solani*; (9) *Blastomyces dermatitidis*; (10) *Coccidiodes immitis*; and (11) *Histoplasma capsulatum*. Of note, case 3 and case 6 were confirmed as *Cryptococcus neoformans* in the same cat. Case 3 was an initial biopsy of multicentric nodular cutaneous lesions that displayed atypical Cryptococcus characterized by few yeasts with no histologically evident capsules and a robust granulomatous inflammatory reaction. Case 6 was taken from the same animal fourteen months later when lesions recurred and progressed to systemic dissemination of typical/classic cryptococcosis characterized in histopathology by myriad yeasts with thick capsules and few reactive macrophages.

### 2.3. Control Cases

Six residual diagnostic samples from animals with no grossly apparent nor diagnosed fungal infections were used to assess the levels of background contamination. These consisted of an oral sample from a dog with peripheral odontogenic fibroma/fibromatous epulis of periodontal ligament origin (22-4384), lymph node tissue from a dog with diffuse large-cell lymphoma (22-7447), a skin biopsy from a dog with grade 2 soft-tissue sarcoma (23-6610), skin biopsies from 2 porcupines with no evidence of dermatophytes (22-9116 with a cutaneous horn and 23-6665 with no gross abnormalities or parasites), and lung tissue from a chicken with minimal lymphoplasmacytic bronchitis (22-11577). Kraken v2.8 was used using a database built from the kraken2-build command option standard to assess the microbial diversity of fungi and protozoa [12].

### 2.4. Amplicon Metagenomic and Bioinformatic Analyses

The analysts working with the FFPE samples were blinded to the case diagnoses. Scrolls of the FFPE tissue from each case were cut with a fresh blade. The Recover All FFPE kit was then used for DNA purification (Thermo Fisher Scientific, Waltham, MA, USA).

Conventional PCR amplification of the fungal nuclear ribosomal 28S rRNA gene variable domain D1 and internal transcribed spacer 1 (ITS1) regions was performed in all cases using primer sets from Tedersoo et al. (2015) [13] (recipe in Table S1). The ITS1 PCR 3-step cycling protocol used was as follows: (1) initial denaturation for 2 min at 95 °C; (2) 35 cycles of 30 s of denaturation at 95 °C, 30 s of annealing at 52 °C, and 30 s of extension at 68 °C; and (3) a final extension for 10 min at 68 °C until a 4 °C hold. The 28S rRNA gene PCR 3-step cycling protocol used was as follows: (1) initial denaturation for 10 min at 95 °C; (2) 35 cycles of 30 s of denaturation at 95 °C, 30 s of annealing at 50 °C, and 60 s of extension at 72 °C; and (3) a final extension for 10 min at 72 °C until a 4 °C hold. Amplification of the full ~5.5 kb rRNA operon (SSU-ITS-5.8S-ITS2-LSU) was attempted only in the first 3 cases, with no success, following Lu et al.’s (2022) protocol [14].

The ATCC Mycobiome Genomic DNA mix (ref: MSA-1010) was used as a positive control. All reactions included negative extraction and amplification controls. The amplicons from conventional PCR were visually confirmed by agarose gel electrophoresis (1.5%, 100 bp DNA ladder Invitrogen cat#15628019, Thermo Fisher Scientific, Waltham, MA, USA) (Figure S1), then purified with magnetic beads (Ampure XP, Beckman Coulter, Brea, CA, USA), and quantified by Qubit (Thermo Fisher Scientific, Waltham, MA, USA). The PCR negative controls for both the amplification of the ITS1 and 28S rRNA gene regions were clear of any detectable bands (Figure S1a,b) and, therefore, not included in further sequencing analyses.

The purified amplicons for each gene target were prepared for sequencing using the Nextera XT library preparation kit (and associated barcodes) and sequenced using the MiSeq instrument with a 2 × 250 bp chemistry cartridge (all from Illumina, San Diego, CA, USA). In order to maximize the compatibility with other Illumina paired-end sequencing chemistries (2 × 150 bp or 2 × 75 bp), enzymatic fragmentation was performed on both targets during the library preparation.

The reads were demultiplexed into individual libraries for each case and target gene (with separate indexes on each) using Illumina’s standard pipeline. The paired reads were concatenated and assessed for their quality and length using FastQC v.0.11.5 [15]. The reference databases SILVA v132 [16] and UNITE v8 [17] were used for the taxonomic markers 28S rRNA gene and ITS1, respectively. All taxonomic assignations were obtained after the database training within the Qiime2 environment with the plugin feature classifier and two different methods for assigning taxonomy: classify-sklearn and classify-consensus-vsearch [18,19,20]. For the latter, different combinations of thresholds were used using the following options: p-min-consensus = 0.75 and p-perc-identity set to 0.90, 0.95, or 0.99. All scripts and databases used are available with annotations at https://github.com/pkmitchell/fungal_metagenomics, accessed on 1 December 2024. The raw data are available at the National Center for Biotechnology Information (NCBI, https://www.ncbi.nlm.nih.gov/) in BioProject: PRJNA556991.

**Table 2:** Best matches for ITS1 and 28S rRNA gene markers analyzed in FFPE tissues in comparison with standard diagnostic testing performed on fresh tissues. Bold text indicates closest match to results of initial standard testing. Each percentage indicates the proportion of quality-filtered sequence reads matching that taxon. ** indicates samples from the same animal (more details in Section 2.2).

Case	Diagnosis in Fresh Tissue	FFPE Tissue ITS Closest Match	FFPE Tissue 28S Closest Match
**1**	*Microsporum canis*	***Microsporum canis* (17%)**	***Arthroderma otae* (synonymous with *Microsporum canis*) 94%**
**2**	*Penicillium* sp., *Exophiala* sp. *(likely E. jeanselmei), Verticillium* sp., and *Rhizopus* sp.	*Cladosporium* (5.5%)	*Capnodiales* (8.5%)
**3**	Atypical cryptococcus neoformans **	***Cryptococcus* (10.2%)**	***Cryptococcus* (6%)**
**4**	*Conidiobolus* spp.	*Cladosporium* (12%)	*Capnodiales* (23%)
**5**	*Aspergillus fumigatus*	***Aspergillus* (18%)**	*Aspergillus lentulus* (1.1%)
**6**	Cryptococcus neoformans var grubii **	***Cryptococcus* (34%)**	*Cryptococcus gattii* (47%)
**7**	*Batrachochytrium dendrobatidis*	*Didymellaceae* (~9%)	***Batrachochytrium dendrobatidis* (8.3%)**
**8**	*Fusarium solani*	***Fusarium* (40%)**	*Hypocreales* (42%)
**9**	*Blastomyces dermatitidis*	***Ajellomycetaceae* (9%)**	*Onygenales* (13%)
**10**	*Coccidiodes immitis*	***Coccidioides* (49%)**	***Coccidioides* (51%)**
**11**	*Histoplasma capsulatum*	***Histoplasma capsulatum* (22%)**	*Onygenales* (83%)

## 3. Results

**Figure 1:**
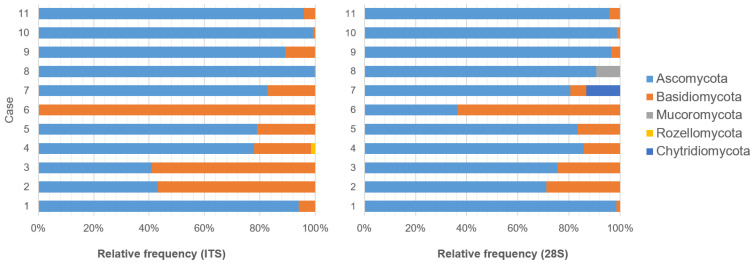
Relative frequencies of fungal phyla observed in FFPE tissues for each gene using the sklearn algorithm.

No amplification of the full-length fungal rRNA was detected in the first three cases. The amplification of ITS1 and 28S rRNA D1 was successful in all cases. None of the control samples (animals with non-fungal pathologies) nor negative process controls yielded a band in the agarose gel electrophoresis. The findings from the FFPE-based short-amplicon metagenomics performed on the fungal cases are summarized in Table 2 and Figure 1. The proportion of reads passing filtration for quality and for chimeras ranged from 72 to 96% for ITS1 and 66 to 89% for 28S rRNA gene D1. Detailed results for ITS are provided in Table S2.

In order to achieve the best matches in Table 2, a combination of different taxonomic identification methods or identity threshold levels (the vsearch approach) was used in the QIIME2 environment (the details of the various bioinformatics methods for the 28S rRNA gene are shown in Table 3). 

Overall, the combination of the ITS1 and 28S rRNA gene results together successfully identified the causative pathogen at the genus level in nine out of eleven cases. The use of ITS1 alone achieved correct genus-level reads in seven out of eleven cases, whereas only four samples could be identified at the genus level with the 28S rRNA gene approach. Moreover, within those four solved cases, only case 7 was resolved only with the 28S rRNA gene approach using the sklearn classifier methods. Interestingly, the other three solved cases, which were also solved via the ITS1 approach, were solved using the vsearch method at 99% identity and not sklearn.

Despite the lack of visual amplification from the control samples, we attempted to sequence their PCR products to ascertain the background microbial compositions in those samples. Due to the much lower number of resulting sequence reads, this was performed using a different analysis method (kraken2). These results are provided in Table S3. Of the reads that could be classified, the 28S products were mostly fungal sequences that were recognizable as commensals (e.g., *Malassezia* sp.), with some human and bacterial reads. The ITS product sequences were largely unclassified, human, or bacterial, with the exception of one control with commensal fungal sequences detected.

## 4. Discussion

The metagenomics and bioinformatics pipelines applied to fresh or frozen samples have been widely demonstrated to powerfully identify a broad array of viruses, bacteria, fungi, and protozoa, including evidence of 913 distinct genomes in one sample of cow rumen [21]. There have been advances in the application of molecular analysis to identify fungi, resulting in the adoption of the ITS1 genetic locus as the primary genetic barcode for fungi [22]. The utilization of metagenomics and bioinformatics has demonstrated markedly diverse fungal populations in fresh environmental samples, with intriguing distinctions between populations at different sites [23]. The application of these and similar techniques to FFPE tissues from the diverse group of host species infected with the varied fungal pathogens we described has not been undertaken before. There are individual case reports or series wherein a single organism was identified, such as *Paracoccidioides* spp. in humans [24], or *Trichophyton*/*Arthroderma*, as we previously described in porcupines [11]. In our prior work, we identified the pathogenic fungus at the genus level in nine of eleven cases and at the family level in one of the two remaining cases and failed to identify the fungal pathogen in just one case [11]. Frickmann et al. applied next-generation sequencing to different tissues containing different fungal lesions, including mucormycosis, histoplasmosis, chromoblastomycosis, rhinosporidiosis, mycetoma, histoplasmosis, and coccidiodomycosis, which resulted in positive fungal pathogen identification in five of seventeen samples [25]. In comparison, the methods we outlined in this work resulted in positive fungal pathogen detection in nine of eleven samples across a broad range of pathogens and hosts. This is similar to a recent study, wherein metagenomic next-generation sequencing and bioinformatics applied to FFPE tissues of pulmonary granulomas from humans revealed fungal or mycobacterial etiologic agents in thirty-six of forty-one samples, with our study expanding the range of host species, tissues, and etiologic agents identified [26].

In this study, we also systematically compared two genetic markers and several bioinformatic strategies for assigning taxonomic identities to the sequencing reads generated using Illumina 2 × 250 bp MiSEQ chemistry. The cost of this study could have potentially been reduced by performing a full library preparation only on the 28S rRNA gene and directly amplifying ITS1 with Illumina adapter sequences. In this configuration, performing library preparations on both amplicons allows us to run on shorter Illumina chemistries for a faster turnaround time. Nonetheless, this can be costly if using the manufacturer-recommended reagent volumes for the library preparations, especially if not batching with other samples/applications. Additionally, subject matter expertise is strongly recommended to assess the taxonomic results in the context of known pathogenic and potentially pathogenic fungi and the clinical presentations.

In general, the application of metagenomics and bioinformatics to identify fungal pathogens in FFPE samples has been limited, with the aforementioned assessment of ITS1 as well as ribosomal RNA genes in the characterization of mucormycosis being notable examples [11,27]. More work has occurred with investigations on viruses and bacteria in FFPE samples, including some specific platforms with specific DNA probes for myriad known viral pathogens and other pathogens in tumor tissues [28], the examination of deeper/mural bacteria in FFPE samples of resected ileum cases of Crohn’s disease [29], longitudinal population assessment through the lens of cancer risk in FFPE samples of gastric tissue infected with *Helicobacter pylori* [30], and the assessment of DNA viruses in FFPE samples from post-transplant lymphoproliferative disorders [31]. By characterizing more loci in addition to ITS1, we demonstrated the effective identification of a varied group of fungal pathogens within varied species and different tissues. Our data indicate that in some rare cases, successful pathogen identification would not have occurred with ITS1 barcoding alone, or it would have occurred with less certainty (cases 7 and 1 in Table 2, respectively). The discrepancies could be due to a number of factors throughout the entire treatment, as described recently in a review regarding the bias in human microbiome studies [32]. Regarding, more precisely, bioinformatics applied to fungal research, high variability in the results was demonstrated while using ITS1 region amplicon sequencing in order to assess plant and soil fungal communities [33]. Additionally, FFPE samples could be damaged by the formalin fixation, as evidenced by the expected lack of amplification of the full rRNA operon (Figure S1c). Specific studies have been completed assessing the alterations and damage to bacterial DNA in FFPE tissues and possible repair strategies [34]. This is a promising future direction for fungal DNA repair for either clinical diagnostics or studies of the mycobiome. While we acknowledge the problems of amplifying relatively long DNA fragments from FFPE material, we believe that an amplicon-sequencing approach is the best available method to tackle fungal diversity in an FFPE sample. Nonetheless, although our shorter amplicons here had successful amplification in all of the cases with fungal pathology, the diagnostic sensitivity and specificity of this method could be affected by other factors, such as the formalin fixation time and exposure to environmental contaminants. In the case of unsuccessful amplification (i.e., no band visualized on a gel), a shotgun metagenomic approach could be the last solution to obtain taxonomic data from said failed PCR samples.

One major advantage of a metagenomic approach is the potential to identify co-infections and non-culturable organisms. In the current study, we identified between two and forty-nine genera of fungi per sample (with at least ten identified reads; see Table S2 for details). Case 11, for example, could only be resolved at the order level by the 28S rRNA approach for the pathogen detected in fresh tissue (*Histoplasma capsulatum*), but the same marker revealed another significant human pathogen, *Mucor irregularis*, in only 0.3% of the reads. Case 8, similarly, harbored 28S rRNA gene sequences of another mucormycosis pathogen, *Lichtheimia ramosa*, at 7.9%. Case 2, a mixed infection in a turtle, could not be resolved here, yet 30 other genera were identified. With animals that may have been in contact with plants and their pathogens, it is expected that a number of sequences from those species will be identified, which was likely the case here. This may also explain the failure of NGS in case 4, a caprine nasal tissue, but in that case, there was a definitive diagnosis made for a single fungal agent. Samples need to be handled with great care in order to not introduce further contamination from commensal fungi of humans (which cannot be ruled out for case 11) or any additional plant material from food or the environment.

Compared with conventional diagnostic methods, metagenomics poses feasibility challenges, particularly to start initially. These include labor for wet and dry lab processes, instrument availability and maintenance, and bioinformatics capabilities for an overall process of approximately one week when the procedure, as described here, is performed. For routine use, laboratories can shorten the time by tailoring to their sequencing platform, e.g., by adding compatible adapters and indexes to their primer sequences. The method provides a large amount of data that need to be interpreted with subject matter and bioinformatics expertise. Even with the limitations of FFPE tissue, it would be difficult to imagine a sample wherein metagenomics would not identify a varied library of fungi, as is evidenced in the capacity for these techniques to parse out differences in fungal communities in different but proximally located soils or to identify over nine hundred organisms in one sample of rumen fluid [21,23]. Implicit in this challenge is a potentially invaluable application of these methods, wherein a study is undertaken examining archived tissues of species affected by an impactful fungus, and FFPE is the lone tissue archive. It is common in veterinary diagnostic laboratories for long-term archival diagnostic samples to consist of FFPE tissues alone. If one were to attempt to identify the presence of an impactful fungal agent, such as *Pseudogymnoascus destructans*, the agent of white-nose syndrome in bats, in archived FFPE tissue prior to the emergence of histopathologic lesions, clinical disease, and population decline, these methods would be ideal. The application of similar methods to those utilized to undertake comparative longitudinal studies of *Helicobacter* sp. populations in gastric biopsies in humans could be adapted to fungi and utilized to assess changes in specific fungi and fungal populations as an impactful disease emerges in a population of animals across a region over multiple years [30]. The currently emerging terbinafine-resistant human pathogen *Trichophyton indotinea sp. nov*. is a timely reminder of the importance of the ecological assessment of fungal pathogens and the utility of advancements in molecular testing for these microbes in archived as well as current clinical specimens [35].

## 5. Conclusions

There is always the possibility that fungi identified via metagenomic approaches, such as amplicon sequencing, in association with a particular known pathogen could be a part of a polymicrobial infection or represent a significant pathogen that is not culturable in standard laboratory settings. The latter indicates the utility and potential wider impacts of the techniques we described to further our understanding and potential treatment of mycoses, especially when only FFPE samples are available. Overall, we demonstrated in this study that FFPE samples can be analyzed with a molecular approach, and we highlighted various more or less successful approaches to tackle fungal diversity in said FFPE samples.

## Figures and Tables

**Table 1:** Sample sources and initial diagnoses. ** indicates samples from the same animal.

Case	Unique ID	Animal	Age (Years)	Sex	Tissue	Diagnosis
1	11-4735C	Dog	7	F	Hairy skin	Folliculitis and furunculosis, multifocal to coalescent, moderate, chronic, pyogranulomatous with dermatophyte fungal hyphae
2	12-4037	Box turtle	13.5	M	Carapace and plastron	Exudative dermatitis, marked, chronic, superficial, heterophilic with mixed fungal hyphae and spores and mixed Gram-positive and Gram-negative bacteria
3	16-438	Cat **	15	CM	Hairy skin	Granulomatous dermatitis with few intralesional yeasts
4	16-4317B	Goat	13	M	Nasal	Rhinitis, mucoid, neutrophilic, and histiocytic with large spore-like organisms
5	16-5386-12	Common loon	Adult	F	Air sac	Mycotic airsacculitis and pneumonia with *Aspergillus fumigatus* organisms
6	17-980N	Cat **	16	CM	Hairy skin	Cryptococcosis
7	18-538B	Gray tree frog	3	F	Skin	Multifocal proliferative and erosive mycotic epidermitis with intralesional chytrid fungi
8	18-10031B	Red-footed tortoise	3	M	Soft tissue/draining tract	Cellulitis, marked, multifocal focally extensive, chronic, necrotizing and granulomatous with fungal hyphae
9	19-1411	Dog	11	F	Popliteal lymph node	Lymphadenitis and cellulitis, pyogranulomatous, severe, chronic, with intralesional fungal yeasts consistent with *Blastomyces dermatitidis*
10	19-3421C	Dog	9	F	Hairy skin	Panniculitis, locally extensive, chronic, pyogranulomatous with numerous fungal spherules consistent with *Coccidioides* spp.
11	19-3965	Cat	13	F	Hairy skin	Dermatitis and cellulitis, focally extensive, chronic, pyogranulomatous with intrahistiocytic fungal spores consistent with *Histoplasma capsulatum*

**Table 3:** Comparison of different taxonomic identification methods (classify_consensus_vsearch and classify-sklearn) and identity threshold cutoffs (90, 95, or 99%) for 28S rRNA gene approaches. The consensus threshold for all vsearch pipelines was fixed at 75%. The detailed taxonomic lineage based on initial standard diagnostics is given above each set of results.

Case	Sklearn	Vsearch 90%	Vsearch 95%	Vsearch 99%
**1**	*Dikarya*; *Ascomycota*; *saccharomyceta*; *Pezizomycotina*; *leotiomyceta*; *Eurotiomycetes*; *Eurotiomycetidae*; *Onygenales*; *Arthrodermataceae*; *Microsporum*; *Microsporum canis*			
	No genera > 5%, 87% *Onygenales*	No genera > 5%, 87% *Onygenales*	No genera > 5%, 87% *Onygenales*	***Arthroderma otae* (synonymous with *Microsporum canis*) 94%**
**2**	Mixed infection			
	No genera > 5%, 8.5% *Capnodiales*	No genera > 5%, 15% *Capnodiales*	No genera > 5%, 15% *Capnodiales*, 9% *Hymenochaetales*	No genera > 5%
**3**	*Dikarya*; *Basidiomycota*; *Agaricomycotina*; *Tremellomycetes*; *Tremellales*; *Cryptococcaceae*; *Cryptococcus*; *Cryptococcus neoformans* species complex			
	No genera > 5%, 12.5% *Capnodiales* + 4.96% *Mearhizium* album, several others between 1 and 5%, 43% *Ascomycota*	No genera > 5%, 36% *Capnodiales*, 9% *Polyporales*	No genera > 5%, *Leucosporidium* 4%, *Capnodiales* 36%, *Polyporales* 9%	*Trametes* 10%, ***Cryptococcus* 6%**, *Leucosporidium* 6%
**4**	*Fungi incertae sedis*; *Zoopagomycota*; *Entomophthoromycotina*; *Entomophthoromycetes*; *Entomophthorales*; *Ancylistaceae*; *Conidiobolus*			
	No genera > 5%	No genera > 5%, *Capnodiales* 23%	No genera > 5%	No genera > 5%
**5**	*Dikarya*; *Ascomycota*; *saccharomyceta*; *Pezizomycotina*; *leotiomyceta*; *Eurotiomycetes*; *Eurotiomycetidae*; *Eurotiales*; *Aspergillaceae*; *Aspergillus*; *Aspergillus subgen. Fumigati*; *Aspergillus fumigatus*			
	*Saccharomycetales* ~18%, no genera > 5%	No genera > 5%, *Saccharomycetales* 39%, *Capnodiales* and *Eurotiales* both >5%	No genera > 5%, *Saccharomycetales* 39%, *Capnodiales* and *Eurotiales* both >5%	No genera > 5%, *Saccharomycetales* 39%
**6**	*Dikarya*; *Basidiomycota*; *Agaricomycotina*; *Tremellomycetes*; *Tremellales*; *Cryptococcaceae*; *Cryptococcus*; *Cryptococcus neoformans species complex*; *Cryptococcus neoformans*; *Cryptococcus neoformans var. grubii*			
	*Cryptococcus gattii* 47%	*Tremellales* 98%	*Cryptococcus gattii* 98%	*Cryptococcus gattii* 98%
**7**	*Fungi incertae sedis*; *Chytridiomycota*; *Chytridiomycota incertae sedis*; *Chytridiomycetes*; *Rhizophydiales*; *Rhizophydiales incertae sedis*; *Batrachochytrium*; *Batrachochytrium dendrobatidis*			
	***Batrachochytrium* 8.3%**, *Pleosporales* 17%, *Saccharomycetales* 9%	No genera > 5%, *Pleosporlaes* 40%, *Saccharomycetales* 20%, *Rhizophydiales* 17%	No genera > 5%, *Pleosporlaes* 40%, *Saccharomycetales* 20%, *Rhizophydiales* 17%	No genera > 5%, *Pleosporlaes* 40%, *Saccharomycetales* 20%, *Rhizophydiales* 17%
**8**	*Dikarya*; *Ascomycota*; *saccharomyceta*; *Pezizomycotina*; *leotiomyceta*; *sordariomyceta*; *Sordariomycetes*; *Hypocreomycetidae*; *Hypocreales*; *Nectriaceae*; *Fusarium*; *Fusarium solani species complex*			
	*Lichteimia* 8%, *Hypocreales* 42%	*Hypocreales* 83%, *Mucorales* 16%	*Hypocreales* 82%, *Mucorales* 16%	*Hypocreales* 82%, *Lichteimia* 14%
**9**	*Dikarya*; *Ascomycota*; *saccharomyceta*; *Pezizomycotina*; *leotiomyceta*; *Eurotiomycetes*; *Eurotiomycetidae*; *Onygenales*; *Ajellomycetaceae*; *Blastomyces*; *Blastomyces dermatitidis*			
	No genera > 5%, *Onygenales* 13%, *Hypocreales* 15%, *Pezizomycotina* 14%, *Saccharomycetales* 5%	*Onygenales* and *Hypocreales* both ~29% *Pezizomycotina* 20%, *Saccharomycetales* 9.5%	*Onygenales* and *Hypocreales* both ~29% *Pezizomycotina* 20%, *Saccharomycetales* 9.5%	*Onygenales* and *Hypocreales* both ~29% *Pezizomycotina* 20%, *Saccharomycetales* 9.5%
**10**	*Dikarya*; *Ascomycota*; *saccharomyceta*; *Pezizomycotina*; *leotiomyceta*; *Eurotiomycetes*; *Eurotiomycetidae*; *Onygenales*; *Onygenaceae*; *Coccidioides*; *Coccidioides immitis*			
	***Coccidioides* 51%**	85% *Onygenales*	97% Onygenales	97% Onygenales
**11**	*Dikarya*; *Ascomycota*; *saccharomyceta*; *Pezizomycotina*; *leotiomyceta*; *Eurotiomycetes*; *Eurotiomycetidae*; *Onygenales*; *Ajellomycetaceae*; *Histoplasma*; *Histoplasma capsulatum*			
	*Onygenales* 47%	83% *Onygenales*	82% Onygenales	82% Onygenales

## Data Availability

All scripts and databases used are available with annotations at https://github.com/pkmitchell/fungal_metagenomics (accessed on 1 December 2024). The raw data are available at the National Center for Biotechnology Information (NCBI; https://www.ncbi.nlm.nih.gov/) in BioProject: PRJNA556991.

## Supplementary Materials

The following supporting information can be downloaded at https://www.mdpi.com/article/10.3390/microorganisms13030533/s1. Figure S1: Agarose gel visualizing conventional PCR amplification in cases and controls. Table S1: PCR primers utilized in this study. Table S2: ITS-identified genera in cases. Table S3: Taxonomic results for control samples without fungal pathology.
