# Supplementary material for: Application of Amplicon Metagenomics to Identify Fungal Pathogens in Formalin-Fixed Paraffin-Embedded Samples: Proof of Concept in Animals with Fungal Pathologies
Source: Microorganisms. 2025 Feb 27;13(3):533. doi: 10.3390/microorganisms13030533 (PMC11944450; doi:10.3390/microorganisms13030533)
Supplement: Supplementary file 1 [file microorganisms-13-00533-s001.zip › microorganisms-3436801-supplementary-Figure S1.pdf]

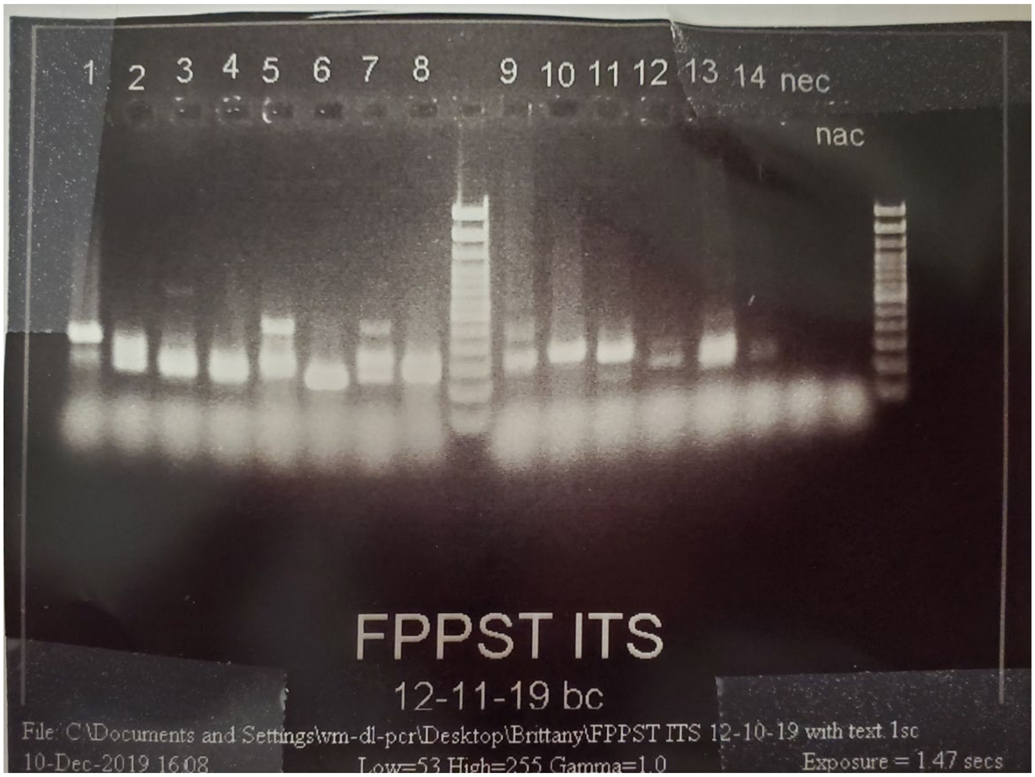
1 2 3 4 5 6 7 8 9 10 11 12 13 14 nec
nac
FPPST ITS
12-11-19 bc
File: C:\Documents and Settings\wm-dl-pcr\Desktop\Brittany\FPPST ITS 12-10-19 with text.1sc
10-Dec-2019 16:08
Low=53 High=255 Gamma=1.0
Exposure = 1.47 secs

(a)

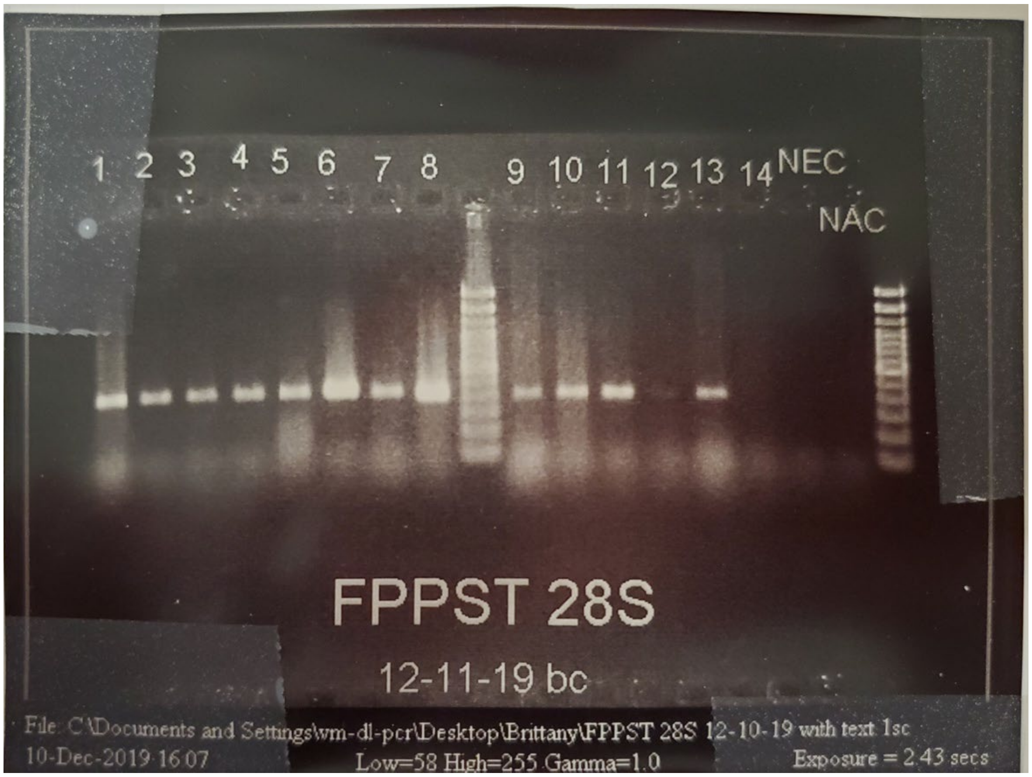
1 2 3 4 5 6 7 8 9 10 11 12 13 14 NEC
NAC
FPPST 28S
12-11-19 bc
File: C:\Documents and Settings\wm-dl-pcr\Desktop\Brittany\FPPST 28S 12-10-19 with text.1sc
10-Dec-2019 16:07
Low=58 High=255 Gamma=1.0
Exposure = 2.43 secs

(b)

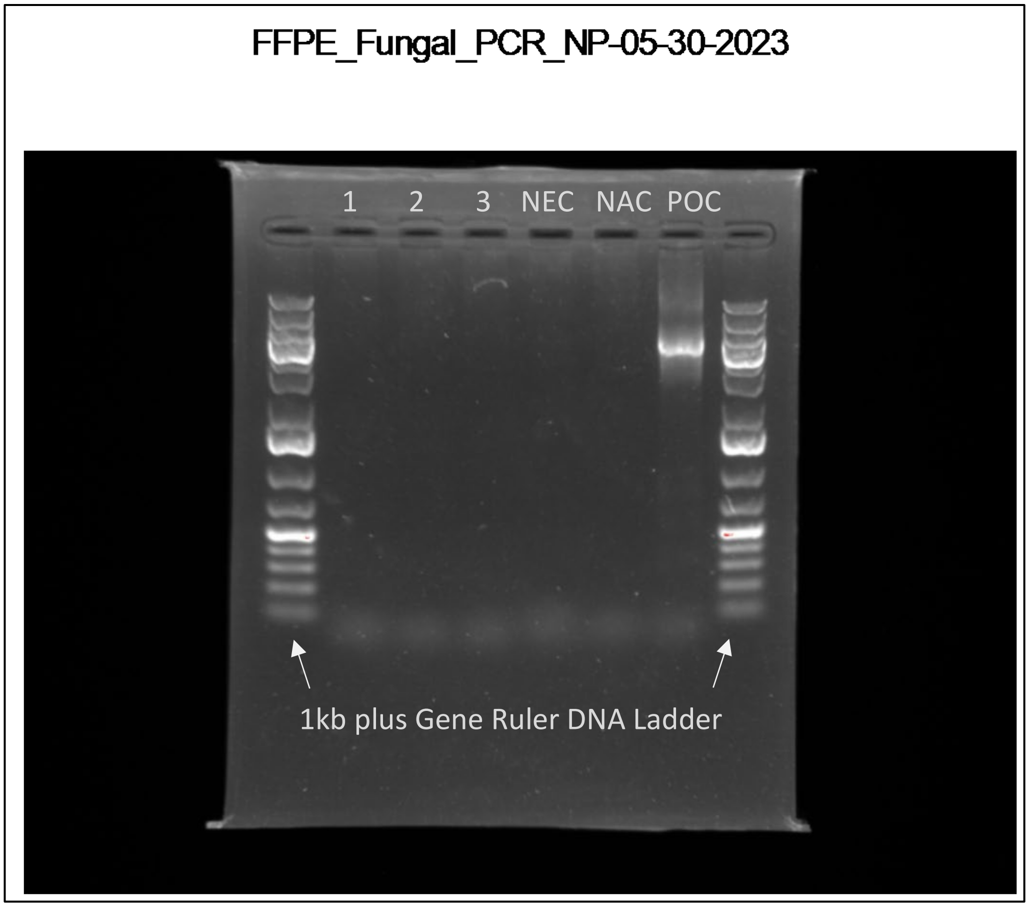

(c)

Figure S1. Gel picture of PCR products after amplification of (**a**) ITS1 targeted region, (**b**) 28S rRNA gene D1 targeted region, (**c**) full fungal 5.5kb rRNA Operon. Columns 1-11 are cases 1-11, samples 12,13 and 14 are not relevant to this study. NEC: Negative Extraction Control. NAC: Negative Amplification Control. POC: Positive Control (ATCC Mycobiome Genomic DNA mix, ref: MSA-1010)
